# Endoscopic obstruction predominantly occurs in right-side colon cancer and endoscopic obstruction with tumor size ≤ 5 cm seems poor prognosis in colorectal cancer

**DOI:** 10.3389/fonc.2024.1415345

**Published:** 2024-06-14

**Authors:** Nong Yu, Shuangming Lin, Xiaojie Wang, Guoxin Hu, Run Xie, Zhipeng Que, Runsheng Lai, Dongbo Xu

**Affiliations:** ^1^ Department of Gastrointestinal Surgery, Longyan First Affiliated Hospital, Fujian Medical University, Longyan, China; ^2^ Department of Colorectal Surgery, Union Hospital, Fujian Medical University, Fuzhou, China

**Keywords:** colorectal cancer, endoscopic obstruction, tumor location, tumor size, disease-free survival

## Abstract

**Background:**

Endoscopic obstruction (eOB) is associated with a poor prognosis in colorectal cancer (CRC). Our study aimed to investigate the association between tumor location and eOB, as well as the prognostic differences among non-endoscopic obstruction (N-eOB), eOB with tumor size ≤ 5 cm, and eOB with tumor size > 5 cm in non-elderly patients.

**Methods:**

We retrospectively reviewed the clinicopathological variables of 230 patients with CRC who underwent curative surgery. The multivariable logistic regression model was used to identify risk factors for eOB. The association between eOB with tumor size ≤ 5 cm and disease-free survival (DFS) was evaluated using multivariate cox regression analysis.

**Results:**

A total of 87 patients had eOB while 143 had N-eOB. In multivariate analysis, preoperative carcinoembryonic antigen (*p* = 0.014), tumor size (*p* = 0.010), tumor location (left-side colon; *p* = 0.033; rectum; *p* < 0.001), and pT stage (T3, *p* = 0.009; T4, *p* < 0.001) were significant factors of eOB. The DFS rate for eOB with tumor size ≤ 5 cm was significantly lower (*p* < 0.001) in survival analysis. The eOB with tumor size ≤ 5 cm (*p* = 0.012) was an unfavorable independent factor for DFS.

**Conclusions:**

The patients with eOB were significantly associated with right-side colon cancer as opposed to left-side colon cancer and rectal cancer. The eOB with tumor size ≤ 5 cm was an independent poor prognostic factor. Further studies are needed to target these high-risk groups.

## Introduction

Colorectal cancer (CRC), with an estimated 1.9 million new cases and 935,000 deaths globally in 2020, stands as the third most prevalent cancer and the second leading cause of cancer-related mortality, contributing to approximately one in 10 cancer cases and deaths (1). Since circa 2010, the incidence of regional-stage and distant-stage disease has increased by about 3% per year in people younger than 50 years and by 2% and 0.5% per year, respectively, in people aged 50–64 years, while rates in people aged 65 years and older have stabilized since about 2015 after a decade of steep decline (2). This trend may necessitate a revision of current screening programs (3).

Endoscopy, particularly colonoscopy, has evolved to be an integral component of the preoperative assessment in patients with colorectal cancer, not just for its diagnostic capabilities but also for its prognostic implications. The occurrence of endoscopic obstruction (eOB) in individuals with colorectal cancer is not uncommon (4), and eOB is defined as the inability of standard colonoscopy to penetrate the tumor, regardless of clinical signs of intestinal obstruction (abdominal distention, peristalsis abdominal pain, nausea, or vomiting) or imaging findings of intestinal obstruction (dilated intestinal loops). Meanwhile, recent studies indicate that eOB is a marker of poor prognosis in patients with stage II colon cancer and stage III rectal cancer following curative surgery (5, 6). Therefore, early identification of eOB is critical for the patient’s treatment. Colorectal obstruction caused by colorectal cancer occurs in 7%–29% of all patients with colorectal cancers (7, 8). Yang et al. indicated that left-side colon cancer was more common than right-side colon cancer in the complete obstructive colorectal cancer compared to the non-obstructive colorectal cancer (9). However, the association between eOB and tumor location has not been investigated. Beyond the influence of tumor location, a distinct variation in tumor size distribution has been noted between eOB and N-eOB. Chalieopanyarwong et al. observed that CRC with N-eOB had a significantly smaller size (4). Smaller tumor size has been reported to be associated with good survival and oncological prognosis in CRC (10). However, several recent studies have reported that tumor size is not associated with survival (11, 12), while others have shown that a smaller size is associated with poorer survival (13, 14). Based on these recent findings, the association between tumor size and oncological prognosis is controversial.

The objectives of this study were to conduct a population-based analysis evaluating the association between primary tumor location and eOB. Additionally, the study aimed to determine the impact of eOB with tumor size ≤ 5 cm on oncological prognosis.

## Materials and methods

### Patient selection

In this single-center retrospective study, patients with histologically confirmed stage I-III colorectal cancer were included. The patient records were maintained in the colorectal tumor database of Longyan First Hospital Affiliated to Fujian Medical University (Fujian, China) between January 2015 and December 2018. The access and use of clinical data were approved by the Institutional Review Board of Longyan First Hospital Affiliated to Fujian Medical University.

**Figure 1:**
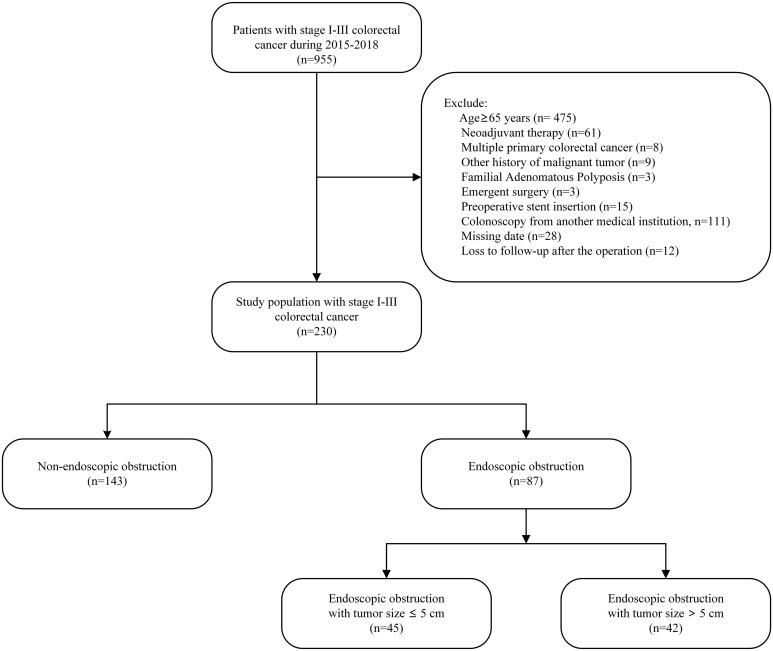
Study flow chart.

All patients who underwent consecutive curative treatments for colorectal cancer were included in this study, with surgical procedures being carried out by a specialized team. Our treatment policy was curative resection of the primary lesion with sufficient margin and appropriate lymph node dissection, followed by observation or adjuvant chemotherapy based on individual risk features. Exclusion criteria were age of 65 years or older, history of neoadjuvant therapy, diagnosis of multiple primary colorectal cancers, other history of malignant tumor, familial adenomatous polyposis, undergoing emergent surgery, preoperative stent insertion, receiving colonoscopy from another medical institution, missing data, and being lost to follow-up post-operation (**Figure 1**).

Tumor stages were coded as described by the 8th edition of the AJCC tumor-node-metastasis grading system (15). The tumor location was classified as the right-side colon (including the cecum, the ascending colon, the hepatic flexure and the transverse colon), the left-side colon (including the splenic flexure and the descending, sigmoid colons and rectosigmoid junction) and rectum (16). The surgical techniques include laparoscopic surgery and open surgery, with the latter encompassing conversion to open surgery. Tumor size was categorized into two groups: ≤ 5 cm and > 5 cm (9). Based on this, the eOB-size group was categorized as N-eOB, eOB with tumor size ≤ 5 cm and eOB with tumor size > 5 cm.

### Statistical analysis

The chi-square test and Fisher’s exact probability test were used to compare categorical data. In order to screen the final predictors of endoscopic obstruction, all candidate predictors with *p* < 0.05 in the univariate analysis were included in the multivariate logistic regression model. In the multivariate analysis, variables with *p* < 0.05 were considered as independent predictors. The Kaplan–Meier method was used to calculate overall survival (OS) and disease-free survival (DFS). And the log-rank test was used to evaluate the difference in survival rate. Univariate and multivariate Cox regression analyses were performed to determine variables related to survival. OS was defined as the time between the date of surgery and the date of death. DFS was defined as the time between the date of surgery and the date of first recurrence of the disease or death. All statistical analyses were carried out using R software (version 4.2.2), and a two-sided *p*-value below 0.05 was considered statistically significant.

## Results

### Clinicopathological characteristics of patients

A total of 87 (37.8%) patients were eOB and 143 (62.2%) patients were N-eOB in the study cohort. 56 (24.3%) patients were below 50 years of age. The collected clinical and pathological characteristics of the patients were subjected to univariate analysis (**Table 1**). Sex, BMI, history of smoking, history of drinking, diabetes mellitus, hypertension, history of abdominal surgery, preoperative carbohydrate antigen 19–9 (CA19–9), differentiation, vascular invasion, perineural invasion and pN stage of eOB patients were similar to those of N-eOB patients. Regarding the patient characteristics, patients with younger age or higher preoperative carcinoembryonic antigen (CEA) had a higher risk for eOB (32.2 vs. 19.6%; *p* = 0.045 and 54.0 vs. 25.9%; *p* < 0.001, respectively). Additionally, the patients who experienced eOB had significantly larger number of harvested lymph nodes (98.9 vs. 88.8%; *p* = 0.010) compared with the patients who experienced N-eOB. Regarding tumor characteristics, the highest rate of right-side colon (51.7%) was observed for patients with eOB. Patients with larger tumor size, higher pT stage and pTNM stage had a higher associated risk of eOB in our study (*p* < 0.001, respectively). For treatment factors, including surgical technique, and adjuvant chemotherapy, there were significant differences between eOB patients and N-eOB patients.

**Table 1:** Clinicopathological characteristics of patients with endoscopic obstruction and non-endoscopic obstruction.

Variable	N-eOB (n=143)	eOB (n=87)	*P* value
Age (years, %)			0.045
<50	28 (19.6)	28 (32.2)	
≥50	115 (80.4)	59 (67.8)	
Sex (%)			0.934
Female	58 (40.6)	34 (39.1)	
Male	85 (59.4)	53 (60.9)	
BMI (kg/m², %)			0.132
<18.5	8 (5.6)	8 (9.2)	
18.5–24.9	91 (63.6)	62 (71.3)	
>24.9	44 (30.8)	17 (19.5)	
History of smoking (%)			0.135
No	99 (69.2)	51 (58.6)	
Yes	44 (30.8)	36 (41.4)	
History of drinking (%)			0.749
No	118 (82.5)	74 (85.1)	
Yes	25 (17.5)	13 (14.9)	
Diabetes mellitus (%)			0.077
No	125 (87.4)	83 (95.4)	
Yes	18 (12.6)	4 (4.6)	
Hypertension (%)			0.324
No	111 (77.6)	73 (83.9)	
Yes	32 (22.4)	14 (16.1)	
History of abdominal surgery (%)			0.625
No	122 (85.3)	77 (88.5)	
Yes	21 (14.7)	10 (11.5)	
Preoperative CEA (ng/ml, %)			<0.001
≤5	106 (74.1)	40 (46.0)	
>5	37 (25.9)	47 (54.0)	
Preoperative CA19–9 (U/ml, %)			1.000
≤37	131 (91.6)	79 (90.8)	
>37	12 (8.4)	8 (9.2)	
Differentiation (%)			0.726
Well/Moderate	133 (93.0)	79 (90.8)	
Poor	10 (7.0)	8 (9.2)	
Harvested lymph nodes (%)			0.010
≥12	127 (88.8)	86 (98.9)	
<12	16 (11.2)	1 (1.1)	
Vascular invasion (%)			0.284
No	103 (72.0)	56 (64.4)	
Yes	40 (28.0)	31 (35.6)	
Perineural invasion (%)			0.319
No	122 (85.3)	69 (79.3)	
Yes	21 (14.7)	18 (20.7)	
Tumor location (%)			<0.001
Right-side colon	19 (13.3)	45 (51.7)	
Left-side colon	39 (27.3)	26 (29.9)	
Rectum	85 (59.4)	16 (18.4)	
Tumor size (cm, %)			<0.001
≤5	117 (81.8)	45 (51.7)	
>5	26 (18.2)	42 (48.3)	
pT stage (%)			<0.001
T1–2	56 (39.2)	6 (6.9)	
T3	54 (37.8)	36 (41.4)	
T4	33 (23.1)	45 (51.7)	
pN stage (%)			0.587
N0	84 (58.7)	47 (54.0)	
N1	34 (23.8)	20 (23.0)	
N2	25 (17.5)	20 (23.0)	
pTNM stage (%)			<0.001
stage I	45 (31.5)	5 (5.7)	
stage II	39 (27.3)	42 (48.3)	
stage III	59 (41.3)	40 (46.0)	
Surgical technique (%)			0.002
Open	10 (7.0)	19 (21.8)	
Laparoscopy	133 (93.0)	68 (78.2)	
Adjuvant chemotherapy (%)			0.005
No	64 (44.8)	22 (25.3)	
Yes	79 (55.2)	65 (74.7)	

In the categorization of patients with eOB, we divided them into two groups based on tumor size: eOB with tumor size ≤ 5 cm and eOB with tumor size > 5 cm. Among the 230 patients reviewed, as illustrated in **Table 2**, 143 (62.2%) were classified as N-eOB, 45 (19.6%) had eOB with a tumor size of ≤ 5 cm, and 42 (18.3%) had eOB with a tumor size of > 5 cm. Follow-up periods were between 10 and 88 months (median, 58 months). A total number of 45 (19.6%) patients had nodal involvement of eOB with tumor size ≤ 5 cm. Age, sex, history of smoking, history of drinking, hypertension, diabetes mellitus, history of abdominal surgery, preoperative CA19–9, differentiation, vascular invasion, perineural invasion, and pN stage were comparable among patients with N-eOB, eOB with tumor size ≤ 5cm, and eOB with tumor size > 5cm. Statistical differences were observed in the population concerning BMI, preoperative CEA, harvested lymph nodes, tumor location, pT stage, pTNM stage, surgical technique, and adjuvant chemotherapy.

**Table 2:** Clinicopathological characteristics of patients with non-endoscopic obstruction, endoscopic obstruction with tumor size ≤ 5 cm and endoscopic obstruction with tumor size > 5 cm.

Variable	N-eOB (n=143)	Endoscopic obstruction with tumor size ≤ 5 cm (n=45)	Endoscopic obstruction with tumor size > 5 cm (n=42)	*P* value
Age (years, %)				0.094
<50	28 (19.6)	14 (31.1)	14 (33.3)	
≥50	115 (80.4)	31 (68.9)	28 (66.7)	
Sex (%)				0.766
Female	58 (40.6)	16 (35.6)	18 (42.9)	
Male	85 (59.4)	29 (64.4)	24 (57.1)	
BMI (kg/m², %)				0.015
<18.5	8 (5.6)	1 (2.2)	7 (16.7)	
18.5–24.9	91 (63.6)	32 (71.1)	30 (71.4)	
>24.9	44 (30.8)	12 (26.7)	5 (11.9)	
History of smoking (%)				0.251
No	99 (69.2)	27 (60.0)	24 (57.1)	
Yes	44 (30.8)	18 (40.0)	18 (42.9)	
History of drinking (%)				0.537
No	118 (82.5)	40 (88.9)	34 (81.0)	
Yes	25 (17.5)	5 (11.1)	8 (19.0)	
Hypertension (%)				0.472
No	111 (77.6)	37 (82.2)	36 (85.7)	
Yes	32 (22.4)	8 (17.8)	6 (14.3)	
Diabetes mellitus (%)				0.108
No	125 (87.4)	42 (93.3)	41 (97.6)	
Yes	18 (12.6)	3 (6.7)	1 (2.4)	
History of abdominal surgery (%)				0.785
No	122 (85.3)	40 (88.9)	37 (88.1)	
Yes	21 (14.7)	5 (11.1)	5 (11.9)	
Preoperative CEA (ng/ml, %)				<0.001
≤5	106 (74.1)	21 (46.7)	19 (45.2)	
>5	37 (25.9)	24 (53.3)	23 (54.8)	
Preoperative CA19–9 (U/ml, %)				0.358
≤37	131 (91.6)	39 (86.7)	40 (95.2)	
>37	12 (8.4)	6 (13.3)	2 (4.8)	
Differentiation (%)				0.194
Well/Moderate	133 (93.0)	43 (95.6)	36 (85.7)	
Poor	10 (7.0)	2 (4.4)	6 (14.3)	
Harvested lymph nodes (%)				0.017
≥12	127 (88.8)	44 (97.8)	42 (100.0)	
<12	16 (11.2)	1 (2.2)	0 (0.0)	
Vascular invasion (%)				0.475
No	103 (72.0)	29 (64.4)	27 (64.3)	
Yes	40 (28.0)	16 (35.6)	15 (35.7)	
Perineural invasion (%)				0.153
No	122 (85.3)	33 (73.3)	36 (85.7)	
Yes	21 (14.7)	12 (26.7)	6 (14.3)	
Tumor location (%)				<0.001
Right-side colon	19 (13.3)	21 (46.7)	24 (57.1)	
Left-side colon	39 (27.3)	16 (35.6)	10 (23.8)	
Rectum	85 (59.4)	8 (17.8)	8 (19.0)	
pT stage (%)				<0.001
T1–2	56 (39.2)	2 (4.4)	4 (9.5)	
T3	54 (37.8)	19 (42.2)	17 (40.5)	
T4	33 (23.1)	24 (53.3)	21 (50.0)	
pN stage (%)				0.172
N0	84 (58.7)	19 (42.2)	28 (66.7)	
N1	34 (23.8)	13 (28.9)	7 (16.7)	
N2	25 (17.5)	13 (28.9)	7 (16.7)	
pTNM stage (%)				<0.001
stage I	45 (31.5)	2 (4.4)	3 (7.1)	
stage II	39 (27.3)	17 (37.8)	25 (59.5)	
stage III	59 (41.3)	26 (57.8)	14 (33.3)	
Surgical technique (%)				0.004
Open	10 (7.0)	9 (20.0)	10 (23.8)	
Laparoscopy	133 (93.0)	36 (80.0)	32 (76.2)	
Adjuvant chemotherapy (%)				0.012
No	64 (44.8)	11 (24.4)	11 (26.2)	
Yes	79 (55.2)	34 (75.6)	31 (73.8)	

### Logistic regression analysis for endoscopic obstruction

To identify predictors of eOB, multivariate analysis was carried out for sample using variables that were available for clinical and pathologic characteristics, including age, preoperative CEA, tumor size, tumor location, and pT stage. Among these factors, preoperative CEA (OR = 2.37; 95% CI: 1.19-4.71, *p* = 0.014), tumor size (OR = 2.56; 95% CI: 1.25-5.24, p = 0.010), tumor location (left-side colon; OR = 0.40; 95% CI: 0.17-0.93, *p* = 0.033; rectum; OR = 0.11; 95% CI: 0.05-0.26, *p* < 0.001), and pT stage (T3; OR = 4.07; 95% CI: 1.43-11.61, *p* = 0.009; T4, OR = 7.45; 95% CI: 2.58-21.55, *p* < 0.001) were found to be independently and significantly correlated with the development of eOB (**Table 3**).

**Table 3:** Logistic regression analysis of clinical and pathological predictors for endoscopic obstruction.

Variable	Reference		Univariable analysis			Multivariable analysis		
			OR	95%CI	*P* value	OR	95%CI	*P* value
Age, years	<50	≥50	0.51	0.28–0.94	0.032	0.88	0.40–1.91	0.742
Sex	Female	Male	1.06	0.62–1.83	0.824	–	–	–
BMI, kg/m²	<18.5	18.5–24.9	0.68	0.24–1.91	0.466	–	–	–
		>24.9	0.39	0.12–1.19	0.099	–	–	–
History of smoking	No	Yes	1.59	0.91–2.77	0.102	–	–	–
History of drinking	No	Yes	0.83	0.40–1.72	0.615	–	–	–
Diabetes mellitus	No	Yes	0.33	0.11–1.02	0.055	–	–	–
Hypertension	No	Yes	0.67	0.33–1.33	0.250	–	–	–
History of abdominal surgery	No	Yes	0.75	0.34–1.69	0.493	–	–	–
Preoperative CEA, ng/ml	≤5	>5	3.37	1.92–5.92	<0.001	2.37	1.19–4.71	0.014
Preoperative CA19–9, U/ml	≤37	>37	1.11	0.43–2.82	0.834	–	–	–
Tumor size, cm	≤5	>5	4.20	2.31–7.64	<0.001	2.56	1.25–5.24	0.010
Tumor location	Right-side colon	Left-side colon	0.28	0.14–0.58	0.001	0.40	0.17–0.93	0.033
		Rectum	0.08	0.04–0.17	<0.001	0.11	0.05–0.26	<0.001
Differentiation	Well/Moderate	Poor	1.35	0.51–3.55	0.548	–	–	–
Vascular invasion	No	Yes	1.43	0.81–2.52	0.224	–	–	–
Perineural invasion	No	Yes	1.52	0.76–3.04	0.241	–	–	–
pT stage	T1–2	T3	6.22	2.43–15.95	<0.001	4.07	1.43–11.61	0.009
		T4	12.73	4.90–33.05	<0.001	7.45	2.58–21.55	<0.001
pN stage	N0	N1	1.05	0.54–2.03	0.881	–	–	–
		N2	1.43	0.72–2.84	0.308	–	–	–

### Survival analysis of overall survival and disease-free survival

**Figure 2:**
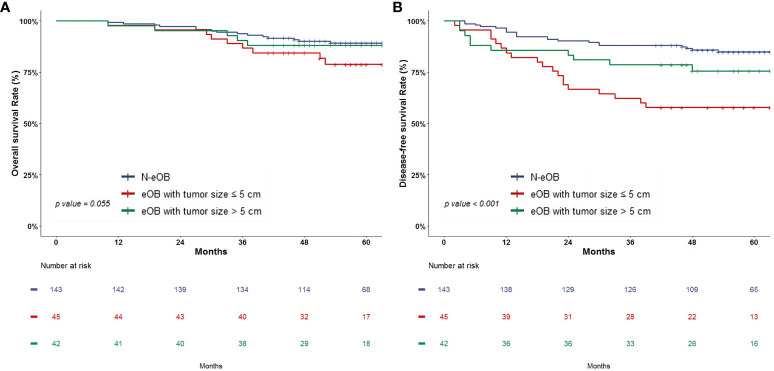
Kaplan-Meier survival analysis of overall survival **(A)** and disease-free survival **(B)**.

There were no significant differences in OS among the three groups: eOB with tumor size ≤ 5 cm, eOB with tumor size > 5 cm and N-eOB (78.8% vs. 88.1% vs. 89.2%, *p* = 0.055) (**Figure 2A**). The DFS rate for eOB with tumor size ≤ 5 cm was significantly lower compared to that of eOB with tumor size > 5 cm and N-eOB (57.8% vs. 75.5% vs. 84.9%, *p* < 0.001) (**Figure 2B**).

### Cox regression analysis for disease-free survival

In the univariate analysis, preoperative CEA, preoperative CA19–9, eOB-size group, differentiation, vascular invasion, perineural invasion, pTNM stage, and adjuvant chemotherapy were all associated with predicting development of DFS (**Table 4**). These variables were included in a multivariable Cox regression analysis. Preoperative CA19–9 (HR = 2.38, *p* = 0.026), eOB-size group (eOB with tumor size ≤ 5 cm vs. N-eOB, HR = 2.32, *p* = 0.012), perineural invasion (HR = 1.97, *p* = 0.040), and pTNM stage (stage III vs. stage I, HR = 6.06, *p* = 0.032) remained significantly associated with DFS. While we did not find that preoperative CEA was statistically significant in the multivariate analysis. Differentiation, vascular invasion and adjuvant chemotherapy were also not found to be significantly associated with worse DFS.

**Table 4:** Cox regression analysis of prognostic predictors for disease-free survival.

Variable	Reference		Univariable analysis			Multivariable analysis		
			HR	95%CI	*P* value	HR	95%CI	*P* value
Age, years	<50	≥50	0.97	0.51–1.86	0.931	–	–	–
Sex	Female	Male	1.00	0.57–1.75	0.990	–	–	–
BMI, kg/m²	<18.5	18.5–24.9	0.74	0.26–2.09	0.569	–	–	–
		>24.9	0.91	0.30–2.74	0.864	–	–	–
History of smoking	No	Yes	1.03	0.58–1.83	0.927	–	–	–
History of drinking	No	Yes	1.14	0.55–2.35	0.721	–	–	–
Diabetes mellitus	No	Yes	0.35	0.08–1.44	0.145	–	–	–
Hypertension	No	Yes	1.32	0.69–2.52	0.403	–	–	–
History of abdominal surgery	No	Yes	1.09	0.49–2.42	0.835	–	–	–
Preoperative CEA, ng/ml	≤5	>5	1.76	1.01–3.06	0.047	1.11	0.61–2.01	0.735
Preoperative CA19–9, U/ml	≤37	>37	2.81	1.37–5.79	0.005	2.38	1.11–5.12	0.026
eOB-size group	N-eOB	eOB with tumor size ≤ 5 cm	3.42	1.84–6.37	<0.001	2.32	1.20–4.48	0.012
		eOB with tumor size > 5 cm	1.78	0.84–3.78	0.134	1.89	0.83–4.32	0.130
Differentiation	Well/Moderate	Poor	2.34	1.05–5.19	0.037	1.89	0.80–4.45	0.147
Harvested lymph nodes	≥12	<12	0.23	0.03–1.67	0.147	–	–	–
Vascular invasion	No	Yes	2.79	1.60–4.86	<0.001	0.99	0.48–2.06	0.986
Perineural invasion	No	Yes	3.51	1.97–6.26	<0.001	1.97	1.03–3.74	0.040
Tumor location	Right-side colon	Left-side colon	0.59	0.28–1.26	0.175	–	–	–
		Rectum	0.75	0.40–1.42	0.380	–	–	–
pTNM stage	stage I	stage II	4.37	0.99–19.36	0.052	2.74	0.54–13.89	0.225
		stage III	10.75	2.58–44.7	0.001	6.06	1.17–31.30	0.032
Surgical technique	Open	Laparoscopy	1.01	0.43–2.38	0.974	–	–	–
Adjuvant chemotherapy	No	Yes	2.36	1.21–4.61	0.012	0.99	0.46–2.13	0.981

## Discussion

Few studies have focused on identifying the factors associated with the emergence of eOB, as well as conducting analyses to evaluate the prognostic implications of eOB with different tumor sizes. This study demonstrated that the incidence of eOB is predominantly higher in the right-side colon compared to the left-side colon in patients diagnosed with non-elderly colorectal cancer. Furthermore, it was noted that eOB with tumor size ≤ 5 cm was associated with a poor survival compared to N-eOB. Conversely, this correlation was not observed for eOB with tumor size > 5 cm.

Unplanned emergency surgeries for colorectal cancer are typically linked with a heightened risk of surgical complications, as well as increased mortality and morbidity rates in emergency scenarios (17–19). A recent study suggested that in instances of luminal obstruction, which is significant enough to impede the further advancement of a colonoscope, physicians should be prompted to contemplate the necessity of urgent surgical intervention, irrespective of the initial symptoms presented (4). Given the importance of preventive interventions, it is crucial to assess the risk factors associated with the emergence of eOB in patients with colorectal cancer. Previous studies presented inconsistent results regarding the relationship between bowel obstruction development and different tumor location. Theoretically, right-side colon cancers are usually ulcerative, and the stool in these regions tends to have a more liquid consistency. Conversely, left-side colon cancers are more likely to present with bowel obstruction, as proliferative lesions are common in this location and the stool is typically of a semisolid consistency. Xinger Lv et al. (20) and Phillips et al. (21) indicated that the left-side colon was more susceptible to bowel obstruction compared with the right-side colon. However, our results demonstrated an increased susceptibility of the right-side colon to eOB, in comparison with the left-side colon and the rectum. Similarly, Kumar et al. observed analogous finding that relatively younger patients present to health center with obstructive colorectal cancer with anatomical shift to the right-side colon (22). In their study, in 54% cases the lesion was in the proximal colon. It is highly plausible that this outcome is attributable to the screening policies. A study indicated that screening for colorectal cancer is associated with lower disease stage (23). Nevertheless, nearly 90% of colorectal cancer patients are diagnosed following the onset of symptoms, exhibiting more advanced disease stages compared to patients identified through asymptomatic screening (24). Furthermore, when presenting with obstruction symptoms, right-side colon cancers are generally at a more advanced stage of progression compared to left-side colon cancers. Relatedly, this study reveals a strong correlation between a more advanced pT stage and the emergence of eOB, which appears to support the hypothesis that eOB is more common in right-side colon cancers. In addition to the reasons previously mentioned, the inability of the colonoscope to smoothly navigate through the space-occupying lesions in the right-side colon, as compared to those in left-side colon and rectum, may also be attributed to discomfort experienced during the colonoscopy or to technical challenges encountered.

In this study, we observed that eOB with tumor size ≤ 5 cm exhibited a poorer DFS, compared to N-eOB. This relationship was present even when controlling for multiple patient-specific prognostic factors, such as preoperative CA19–9, perineural invasion, pTNM stage. Therefore, to give a potential explanation of our findings, we hypothesized that eOB with tumor size ≤ 5 cm may be a surrogate marker for biological aggressiveness resulting in inferior DFS, which indicated that initial biological heterogeneity of colorectal cancers determined their distinct growth pattern and different invasive and metastatic abilities. In this study, eOB with tumor size ≤ 5 cm was associated with higher pT stage, reflecting a vertical growth pattern. Tumors with a vertical growth pattern may have early acquired high metastatic potential which enable them to breach basal membrane, invading the surrounding tissue and finally disseminating to regional lymph nodes and distant metastasis (25). In contrast, tumors with a horizontal growth pattern reflected by eOB with larger tumors underline a biologically indolent disease and a lower metastatic ability. The fact that clinicians are more likely to treat large tumors more aggressively may also explain our results. Multivisceral resection (MVR) is associated with increased tumor size in locally advanced colorectal cancer (26). In addition, the larger tumor size often leads to more complete lymph node resection and evaluation in colorectal cancer (27). These more aggressive treatments may result in better survival rates. Therefore, in the diagnosis and treatment of colorectal cancer, the evaluation of eOB with smaller tumors should not be overlooked. In addition, preoperative CEA was not an independent prognostic biomarker according to our study. One study has shown that patients with elevated preoperative CEA that normalizes after surgery have a similar outcome to patients with normal preoperative CEA (28). This adequately demonstrates the limitations of preoperative CEA in predicting postoperative recurrence.

This study indeed has several limitations. (1) This study was conducted as a single-center retrospective analysis. Consequently, the number of patients was limited, and the selection process adhered to stringent inclusion and exclusion criteria. This approach may have introduced potential selection bias. (2) The stratification by tumor size and eOB led to relatively small subgroups, which reduced the statistical power to discriminate small differences. (3) Neoadjuvant chemotherapy, radiotherapy and laboratory examinations were not included in the present study. Further investigations of multi-center prospective study should be conducted and more baseline characteristics should be enrolled.

## Conclusion

Among non-elderly patients, those with eOB were significantly associated with right-side colon cancer as opposed to left-side colon cancer and rectal cancer. The eOB with tumor size ≤ 5 cm was associated with lower DFS, while this association was not observed for eOB with tumor size > 5 cm. The observed shift in the incidence of eOB towards the right-side colon, coupled with the result that eOB with tumor size ≤ 5 cm may denote a more aggressive form of malignancy, highlights the imperative for comprehensive research.

## Data availability statement

The raw data supporting the conclusions of this article will be made available by the authors, without undue reservation.

## Ethics statement

The studies involving humans were approved by The Ethical Review Board of the Longyan First Affiliated Hospital of Fujian Medical University (approval number: LYREC2024-k024-01). The studies were conducted in accordance with the local legislation and institutional requirements. The ethics committee/institutional review board waived the requirement of written informed consent for participation from the participants or the participants' legal guardians/next of kin because this study utilizes medical records obtained from previous research and meets all of the following conditions: Previous research has obtained written consent from the participants, allowing other research projects to use their medical records or specimens. This research complies with the permission conditions of the original informed consent. The confidentiality of the participants' privacy and identity information is ensured.

## Author contributions

NY: Data curation, Methodology, Software, Writing – original draft. SL: Conceptualization, Data curation, Writing – original draft. XW: Writing – review & editing, Conceptualization. GH: Data curation, Methodology, Writing – original draft. RX: Data curation, Writing – original draft. ZQ: Data curation, Writing – original draft. RL: Data curation, Writing – original draft. DX: Conceptualization, Writing – review & editing, Data curation, Methodology.
